# Book Review

**Published:** 1936

**Authors:** 


### Reviews of Books

**The Medical Dictator.** By Major Greenwood, F.R.S., D.Sc., F.R.C.P. Pp. 214. Illustrated. London: Williams and Norgate. 1936. Price 7s. 6d.—This is an interesting series of biographical sketches of various medical men, some perhaps to be accounted great by any criteria in the eyes of all, some only great to a more limited and discerning clientele, and some only, perhaps, great to their friends. Dr. Greenwood (the "Major" is only an unusual Christian name, and is not indicative of any military rank), as the medical statistician, would be inclined to include some people of his own speciality, who are less well-known to the ordinary man in the street, hence the appreciation of William Farr, the originator of accurate medical statistics in England, and of Louis, the exponent of the "Numerical Method." Both of these are interesting sketches. The one we liked least was the most erudite—that of Galen. Two old-time British physicians, John Freind of the seventeenth and eighteenth centuries and Latham of the nineteenth, are interestingly sketched. The other two portraits drawn are of Sir William Osler, most certainly one of the most lovable of men, and of Arthur William Bacot. One suspects that the *raison d'être* of the whole of this little book is the author's desire to pay homage to the memory of the last-named, a close personal friend of his own. He was a business man whose hobby was entomology, and who eventually was able to take up that branch of science as his life work, performing invaluable work upon the transmission of plague by the rat flea, and during the war upon louse-borne diseases, as trench-fever and typhus. He himself fell a victim to the last-named disease during his researches in Egypt. This is by far the best of the sketches, there being a warm personal note necessarily lacking in the others. The mere mention of a medical statistician calls to mind a sort of human ready-reckoning machine, so that one's heart warms to a human note in the most distinguished British member of the class. He seems to have but a poor impression of the mentality, receptivity and even general intelligence of the Fellows of the Royal College of Physicians of past days, and of the rules and regulations of the College itself. It is an interesting speculation to wonder what the late lamented fellows would have thought and said of a modern F.R.C.P. who thus lightly esteems and even gently ridicules the traditions and history of that august foundation.

**British Masters of Medicine.** Edited by SIR D'ARCY POWER, K.B.E., F.R.C.S. Pp. xvi., 242. Illustrated. London: Baillière, Tindall & Cox. 1936. Price 7s. 6d.—This series of twenty-four short biographies by various writers appeared originally in the *Medical Press and Circular* during the years 1934 and 1935. Many of the subjects have been written about frequently, for example Harvey, Sydenham, Hunter and Jenner; but there are new comers also, John Floyer of Lichfield, by whose advice Samuel Johnson was taken to be touched for the Evil by Queen Anne, and who wrote on cold bathing, the rate of the pulse and respiration, on blood volume, and ascribed the dyspnœa of asthma to constriction of the bronchi. Robert Willan is another little-known master whose life history is worth preserving. Recognition is also given to many modern masters. William Turner, the Edinburgh anatomist and great administrator, Hugh Owen Thomas and his illustrious disciple Robert Jones, together with Manson, Osler, James Mackenzie and Starling. It is well to be reminded that there have been giants even in our own times. For the biographies we have nothing but praise; they are short, readable and full of interest.

**Applied Physiology.** Sixth Edition. By SAMSON WRIGHT, M.D., F.R.C.P. Pp. xxxii., 686. Illustrated. London: Oxford University Press. 1936. Price 20s.—The sixth edition of this work will need no recommendation to the vast majority of medical practitioners or students. The fact that six editions have been published in the ten years that have elapsed since this invaluable book was first published is sufficient testimony, if any were needed, to its well-deserved popularity. Physiology is of necessity the basis of all medical work, and any practitioner who wishes to keep abreast of current thought and ideas must maintain his interest in physiology. The new edition is some seventy pages larger than its predecessor on account of the large amount of fresh matter that has had to be included, and in addition many sections have been entirely rewritten in order to bring the whole completely up to date. In this, as in all the previous editions, the clinical significance of the facts described is constantly stressed. The book is well produced and there is an excellent index. It is a work which could be read with benefit not only by every student but by every practitioner of medicine. To anyone contemplating a higher degree in either medicine or surgery it is simply "essential."

**Asthma, Hay Fever and Migraine.** By A. G. AULD, M.D. Pp. x, 257. London : H. K. Lewis & Co. 1936. Price 12s. 6d. —Dr. A. G. Auld has collected into a single volume a number of clinical lectures and articles which he has published in various journals since 1885. The majority of these deal with Allergy in its different aspects ; that is to say, Asthma, Hay Fever and Migraine form the chief part of the volume. Readers will probably find these chapters the most interesting, since the author relates at some length his experiences with peptone injections, pyrogenic therapy by means of colloidal metals and other methods, and the treatment of hypersensitiveness in various ways. Some of his reprinted papers are only interesting because they show Dr. Auld to have been almost before his time in his physiological approach to the study of medicine. Nowadays every physician must of necessity be primarily a physiologist, but in the eighteen eighties and nineties such an attitude was uncommon. Dr. Auld reprints a very foolish article on "The Roentgen Rays in the Diagnosis of Pulmonary Disease," belittling the value of their aid in physical examination of the chest. The article was written in 1903, and there is nothing to indicate that the author has changed his views or marched with the times, unless the omission of all reference to this article in the index betokens a desire to disown a youthful indiscretion. It is a pity that the proof-reading has been so badly done. Small errors in spelling are irritating and there are too many of them, one or two may be oversights, but the repetition of "intra muscularily" for "intra muscularly" occurs nine times, "collodial" for "colloidal" twice, and there are in addition at least eighteen other mistakes in spelling or type-setting, none of which ought to have escaped the publishers' proof-reader.

**Endocrine Organs in Health and Disease.** By Sir HUMPHRY DAVY ROLLESTON, Bart., M.D., D.Sc., D.C.L., LL.D. Pp. xii, 521. Illustrated. London : Oxford University Press. 1936. Price 36s.—It cannot be often in these days of rapid progress in medical science that a book of this scope is written by a single author. The title may sound sufficiently comprehensive ; but it is not necessary to read more than two or three chapters to realize how modest that title is. The book is the outcome of the author's 1933 and 1934 Fitzpatrick Lectures on the "History of the Endocrine Organs," and history is its chief interest. Each chapter is devoted to one of the endocrine glands, and commences with a remarkably full historical review of its structure and function. At times the historical sections may to some tend to become somewhat bald (this is no fault of the author's) but they are pleasantly relieved here and there by miniature biographies, principally of nineteenth-century physicians and others who played important parts in the development of our knowledge of the glands in question. The historical portion of each chapter is followed by descriptions of the abnormalities and syndromes associated with the organ, their ætiology, diagnosis and treatment, each part with its own selected bibliography. The result is a remarkably comprehensive account of past and present-day endocrinology. The book has been brought as much up-to-date as possible since the delivery of the Lectures, and one cannot but admire the way in which the author has so successfully dealt, for example, with the pituitary and the gonads in our knowledge of which such rapid advance is now being made. However, the general reader will probably be most struck by the masterly review of the pioneers and their discoveries in the various branches of the subject to which Sir Humphry Rolleston's historical taste has done full justice. There is a good index and the book is well printed.

**The Anæmias.** By JANET M. VAUGHAN, D.M., M.R.C.P. Second Edition. Pp. xiv., 309. Illustrated. London : Oxford University Press. 1936. Price 12s. 6d.—In view of the rapid progress which is being made in the science of hæmatology, it is by no means too soon to welcome a second edition of this useful book. The authoress does not claim that any great discoveries have been made since 1934, but much work that was then hypothetical has been confirmed and consolidated. The scope and standard of the book are definitely beyond that required by the medical undergraduate, but it is eminently suitable for those working for higher degrees, for the pathologist and clinician alike. Throughout, there is a close correlation of the medical and pathological aspects of diseases ; not only the blood picture and morbid anatomy, but the ætiology, treatment and differential diagnosis are fully dealt with. The classification of anæmias, the chapters on the leuco-erythroblastic anæmias, and megalocytic hyperchromic anæmias associated with gastro-intestinal lesions, and the comprehensive bibliography are of particular interest. Diagrams and illustrations are clear, and, within the scope of the book, adequate, and, apart from one or two trifling errors, the text is lucid and interesting. Theories are put forward logically and persuasively, but without dogmatism, and the book can be cordially recommended to those interested in blood diseases.

**A Short Practice of Surgery.** By HAMILTON BAILEY, F.R.C.S., and R. J. MCNEILL LOVE, M.S., F.R.C.S. Third Edition. Pp. viii., 995. Illustrated. London: H. K. Lewis & Co. 1936. Price 28s.—The appearance of another edition of this work within a year bears eloquent testimony to its popularity among medical students. It is the most succinct and comprehensive guide to surgery. The style is interesting and lucid, and frequent classifications interspersed in the text afford valuable aids to memory. The book is well furnished with information on the recent advances in procedures such as blood transfusion, cystoscopic work and bone and joint surgery. The closing chapter on whitlows and their complications is a masterpiece in clarity of press and picture. There is still some room for improvement even in this splendid production. Some of the orthopædic pages present the treatment of the renowned problems of deformities in a conventional and optimistic way which is apt to mislead. For instance talipes, hallux valgus, etc., still defy and embarrass even the experts. It would be cause for congratulation of the authors if such inveterate problems were given more adequate attention. The necessary space for such alterations might be gained without lengthening the volume if the student were spared the discussion on the delayed treatment of appendicitis —a dangerous sphere for any but the consulting surgeon— and the story of the hydatid cyst. This last is such a rarity in practice that it is better left to post-graduate works or to the pathologist. Despite its drawbacks, which are not peculiar to this textbook, there is no doubt that the student who masters it will please both examiner and patient.

**Vascular Disorders of the Limbs.** By Sir THOMAS LEWIS, F.R.S., M.D., D.Sc., LL.D., F.R.C.P. Pp. xii., 111. London: Macmillan & Co. 1936. Price 6s. 6d.—This book is a very valuable presentation of our present knowledge of circulatory disturbances in the limbs, in elucidating which Sir Thomas

Lewis and his colleagues at University College Hospital have played such a large part. The various vascular disorders that may affect the limbs are described accurately and concisely, and the reader is shown how to investigate and treat these conditions without the use of any more elaborate piece of apparatus than a sphygmomanometer, and the " dorsal surface of the middle phalanx of the flexed finger." The latter, we are told, is the best instrument for comparing the temperature of two limbs. Starting with a description of the anatomy and physiology of the circulation in the limbs, the author proceeds to describe the various obliterative and vascular diseases that may be met with and their differentiation and treatment. This is a book to be read by all students and practitioners of medicine, whether they be in general practice or consulting medicine or surgery. In addition, it is a beautiful example of the value of accurate and carefully controlled clinical observations, *i.e.* of true " scientific " medicine.

**The Relief of Pain.** By HAROLD BALME, M.D., F.R.C.S. Pp. xvi., 392. London : J. and E. Churchill. 1936. Price 12s. 6d.—The author devotes the earlier chapters of his book to general considerations of the problems of pain, considered in the light of physiology and anatomy. He then passes on to an attempt to find a classification of types of pain, a difficult thing to do, but obviously useful clinically. Then follows a detailed consideration of individual problems in treatment, with carefully considered therapeutic suggestions. He leaves hardly any clinical field out of his purview. In the latter part of the book there are sections on balneology and physiotherapy by Dr. Matthew Ray, which should interest the uninitiated. The author displays considerable enthusiasm for short and ultra-short wave therapy, and the views expressed are well worthy of consideration by many to whom this method of treatment is new. The book closes with a useful chapter on the analgesic drugs, notably the modern unofficial preparations giving their composition and indications for their use. For purposes of reference, and as an aid in dealing with the everyday problems of practice, the book may be strongly recommended to general practitioner and specialist alike.

**Squint Training.** By M. A. PUGH, M.R.C.S., L.R.C.P. Pp. viii., 117. Illustrated. London : Oxford University Press. 1936. Price 7s. 6d.—The subject of orthoptic training for squint and errors of ocular muscle-balance has aroused an increasing amount of interest in recent years, and a book describing modern methods of squint training was much needed. Dr. Pugh, medical officer in charge of the Orthoptic Department at Moorfields Hospital, has in this volume produced an excellent guide to the subject. The preface states that the book is intended to give only " a practical classification of cases, an accurate method of measuring the deformity, and the technique of treating a squint," and while it admirably achieves these objects, its value is greatly enhanced by additional chapters on the selection of cases for operation, post-operative results, and the results of various methods of treatment. A useful appendix contains illustrated descriptions of a number of modern training instruments. To all engaged in ophthalmic work, including especially members of the School Medical Service concerned with the question of treating squint, this book can be recommended as well worth studying.

**A Manual of Practical Obstetrics.** By O'DONEL BROWNE, M.B., B.Ch., F.R.C.P.I. Pp. vi., 363. Illustrated. Bristol: John Wright & Sons. 1936. Price 20s.—The Manual gives a short account of the methods used in practical obstetrics in Dublin. The scope of the matter is very full, even to a description of jejunostomy for hyperemesis, which is of very doubtful value. The Dublin method for the treatment of eclampsia is described in full, while Stroganoff's method is dismissed in a few lines. The whole book is of very doubtful value ; it is too sketchy as a text-book, and not concise enough as a " cram " book, to be of use to students, but would be of value to general practitioners who employ the Dublin methods.

**A Text-Book of Midwifery in the Tropics.** Second Edition. By V. B. GREEN-ARMYTAGE, M.D., F.R.C.P., and P. C. DUTTA, M.B., F.R.C.S. Pp. xiv., 448. Illustrated. London: Butterworth & Co. (India), Ltd. 1936. Price 12s.—This text-book is written in the style of the popular " Synopsis " series, and is, in fact, a synopsis of midwifery rather than a text-book. The principles of the practice of obstetrics do not differ whatever the climate may be, and so the teaching we find is the orthodox, and applicable to any country. If used in conjunction with an ordinary text-book, it should be of great value to the student for examination purposes, and to the practitioner whether practising in the British Isles or the tropics.

